# Crystal Structure of KLHL3 in Complex with Cullin3

**DOI:** 10.1371/journal.pone.0060445

**Published:** 2013-04-03

**Authors:** Alan X. Ji, Gilbert G. Privé

**Affiliations:** 1 Department of Biochemistry, University of Toronto, Toronto, Ontario, Canada; 2 Department of Medical Biophysics, University of Toronto, Toronto, Ontario, Canada; 3 Ontario Cancer Institute, Campbell Family Institute for Cancer Research, University Health Network, Toronto, Ontario, Canada; George Washington University, United States of America

## Abstract

KLHL3 is a BTB-BACK-Kelch family protein that serves as a substrate adapter in Cullin3 (Cul3) E3 ubiquitin ligase complexes. KLHL3 is highly expressed in distal nephron tubules where it is involved in the regulation of electrolyte homeostasis and blood pressure. Mutations in KLHL3 have been identified in patients with inherited hypertension disorders, and several of the disease-associated mutations are located in the presumed Cul3 binding region. Here, we report the crystal structure of a complex between the KLHL3 BTB-BACK domain dimer and two copies of an N terminal fragment of Cul3. We use isothermal titration calorimetry to directly demonstrate that several of the disease mutations in the KLHL3 BTB-BACK domains disrupt the association with Cul3. Both the BTB and BACK domains contribute to the Cul3 interaction surface, and an extended model of the dimeric CRL3 complex places the two E2 binding sites in a suprafacial arrangement with respect to the presumed substrate-binding sites.

## Introduction

Targeted ubiquitination can direct substrate proteins to a variety of functional fates, including proteosomal degradation, the modulation of protein interaction networks, and altered subcellular localizations. The largest class of E3 ligases are the Cullin-RING Ligases (CRLs), which are further identified according to the type of cullin chain that constitutes the central scaffolding unit [1], [2]. For example, the CRL3 complex is built around a Cullin3 (Cul3) component. While many CRL complexes interact with bipartite substrate adaptor proteins, such as the Skp1/F-Box adaptor protein complexes in CRL1, CRL3s differ by forming a complex with single-chain substrate adapters that bind directly to both the cullin and substrate [3]–[6].

Many of the known CRL3 substrate adapters belong to the BTB-BACK-Kelch family of proteins. These proteins are made almost entirely of three concatenated structural domains: the BTB and BACK domains form the platform that engages the N-terminal region of Cul3, while the Kelch repeat domain forms a β-propeller structure for substrate binding. In humans, most of the 52 BTB-BACK-Kelch proteins can be classified into two named groups, namely the 39 KLHL proteins and the 11 KBTBD proteins [7]–[9]. Notable proteins from this family include Keap1 (KLHL19), an electrophile-sensing regulator of Nrf2 [10]–[12], KLHL9, which is associated with an autosomal distal myopathy [13], KLHL12, a regulator of the dopamine D4 receptor [14] Dishevelled [15], [16] and CPOII coat function [17], KLHL20, a regulator of hypoxia-inducible factors [18], [19], and KLHL3, a regulator of hypertension with mutations identified in pseudohypoaldosteronism type II (PHAII) [20]–[22].

PHAII, also known as familial hyperkalemic hypertension, is a rare autosomal dominant disease characterized in part by elevated electrolyte and reduced bicarbonate levels in the blood. The characterization of the molecular defect in this disease has provided key insights into the mechanisms of blood pressure regulation [23]–[27]. Exome sequencing of affected individuals has established that PHAII can be caused by mutations in either KLHL3 or Cul3, and these mutations are correlated with an increased activity of the NaCl cotransporter (NCC) in the distal convoluted tube (DCT) [21], [22]. Because E3 ligases can regulate the endocytosis of integral membrane proteins [28]–[33], KLHL3 may regulate electrolyte homeostasis by regulating NCC trafficking via CRL3^KLHL3^-dependent ubiquitination.

Here, we report the crystal structure of a KLHL3^BTB-BACK^/Cul3^NTD^ complex and characterize the Cul3 binding properties of a series of KLHL3 PHAII mutations. We previously reported the structure of the SPOP^BTB^/Cul3^NTD^ complex [9]. In addition, a structure of KLHL11^BTB-BACK^ in complex with a Cul3 N-terminal domain has been recently reported [34]. An analysis of the three available BTB/Cul3 complexes provides insight into how Cul3 is able to bind to a large number of different adaptor proteins. The structure of the KLHL3^BTB-BACK^/Cul3^NTD^ complex allows an expanded comparison of the BTB-BACK/Cul3 binding interface and allows a more accurate modeling of other intensely studied BTB-BACK-Kelch proteins such as Keap1.

## Materials and Methods

### Cloning, Protein Expression and Purification

An expression construct for human KLHL3 comprising residues 27–276 (KLHL3^BTB-BACK^) was designed using the web-based Crystallization Construct Designer [35] and cloned into a pMCSG7 vector via ligation independent cloning [36], producing a protein with an N-terminal 6His tag. The version of KLHL3^BTB-BACK^ used in crystallization was further modified by surface entropy reduction (SER). The Surface Entropy Reduction prediction (SERp) web server [37] identified K87, K89 and K90 as three nonconserved residues predicted to be exposed at the protein surface. These three lysines were mutated to alanine residues by PCR mediated site directed mutagenesis for the protein used in crystallization. The KLHL3^BTB-BACK^ protein used in the solution-based experiments did not include the SER mutations, and the PHAII mutations were introduced into the wild-type KLHL3^BTB-BACK^ expression plasmid by PCR mediated site directed mutagenesis. The N-terminal domain of Cul3 comprising residues 20–381 incorporating the stabilizing mutations I342R/L346D (Cul3^NTD^) was cloned into a pET32a vector as described previously [9].

KLHL3^BTB-BACK^ and Cul3^NTD^ were expressed separately in *E. coli* BL21 DE3 Codon+ cells. Cultures were grown at 37°C to an OD600 of 0.8. The temperature was then reduced to 15°C and the cultures were induced with 1 mM IPTG and grown overnight. Cells were harvested, lysed and the His-tagged proteins were purified by metal ion chelate chromatography on NiNTA resin. The N-terminal thioredoxin-His tag on the Cul3^NTD^ protein was removed with TEV protease. The final purification step for both proteins was size exclusion chromatography on a Superdex S75 column in 20 mM Tris pH 7.5, 150 mM NaCl, 1 mM TCEP, and 10% v/v glycerol (buffer A).

### Crystallization, Data Collection, Structure Solution and Refinement

Crystals of the KLHL3^BTB-BACK^/Cul3^NTD^ complex were grown by hanging drop vapor diffusion at room temperature. KLHL3^BTB-BACK^ and Cul3^NTD^ were mixed in a 1∶1 molar ratio at a total protein concentration of 10 mg/ml. Hanging drops were set by mixing 1 µl of the protein solution with 1 µl of a reservoir solution containing 0.1 M sodium tartrate and 17% w/v PEG 3350, and incubating against 1 mL of reservoir solution. Crystals were soaked in a reservoir solution containing 15% ethylene glycol for 30 s prior to flash freezing. Diffraction data were collected at 100 K on beamline 19-ID at the Advanced Photon Source at a wavelength of 0.98 Å using ADSC Quantum 315r detector. Data were processed with HKL3000 [38], [39]. The KLHL3^BTB-BACK^/Cul3^NTD^ structure was solved by molecular replacement with PHENIX AutoMR [40] using a search model based on a KLHL11/Cul3 complex (PDB ID 4AP2) [34]. The top solution placed one chain of KLHL3^BTB-BACK^ and one chain of Cul3^NTD^ in the asymmetric unit, with a solvent content of 66%. The solution generated a KLHL3 BTB-BTB homodimer via a crystallographic 2-fold symmetry operator, even though no information about this expected dimer was included in the molecular replacement procedure. As a further test of the solution, an independent molecular replacement search was carried out using the same search model, except that residues corresponding to helices H1 and H2 of Cul3 were deleted. Following rigid body refinement of this partial model, difference density maps clearly showed the density for the deleted residues (Figure S1A). Similarly, a search model based on the SPOP^BTB^/Cul3^NTD^ (PDB ID 4EOZ) [9] gave an equivalent solution. In this case, positive difference density was observed for KLHL3 helices in the BACK domain, despite the fact that residues in this region were not present in the search model.

Refinement and model building were performed using PHENIX [40] and Coot [41]. Ramachandran dihedral restraints were used in the final stages of refinement. The final model consisted of residues 32–223 of KLHL3^BTB-BACK^ and residues 26–379 of Cul3^NTD^. The quality of the final model was verified with a composite omit map calculated with PHENIX [40] (Figure S1B). Structural superpositions and renderings were performed in PyMol [42]. KLHL3^BTB-BACK^/Cul3^NTD^ interface residues and buried surface area were determined using the EMBL PISA web server [43]. Transformation functions for BTB dimer and BTB-Cul3 interfaces were calculated using EMBL Dali Lite web server [44], [45]. Atomic coordinates and structure factors have been deposited in the Protein Data Bank under accession code 4HXI.

### Size Exclusion Chromatography

Protein samples were injected onto a Superdex S75 size exclusion column equilibrated with buffer A and elution was monitored at 280 nm.

### Isothermal Titration Calorimetry

Isothermal titration calorimetry (ITC) binding experiments were performed using a VP-ITC Micro Calorimeter at 25°C. All proteins were dialyzed in buffer A for three days prior to analysis. Aliquots of KLHL3^BTB-BACK^ at 150 µM were injected into Cul3^NTD^ solutions at 15 µM. Data were processed using Origin, and binding isotherms were calculated based on a one-site binding model. A single titration was conducted for wild type KLHL3 BTB-BACK and each mutant. K_d_ error values are based on the sum of square deviations between the non-linear regression curve and the experimental data.

### Homology Modeling

A model of the complete SCF3^KLHL3^ ubiquitin ligase complex was generated following the approach used in making the SCF3^SPOP^ model [9]. The complex is based in part on the known structures of Cul1-Rbx1-Skp1-Skp2 [4]. The Cul1 chain was replaced by a full length Cul3 model in which the C-terminal domain was based on Cul1. The E2 Ubch7 was positioned onto Rbx1 by superposing the RING domains from Rbx1 and c-Cbl from the c-Cbl-Ubch7 complex [46]. Ubiquitin was positioned onto Ubch7 by superposing E2–24 from the E2–24-ubiquitin complex on Ubch7 [47]. I-Tasser [48] was used to generate Keap1^BTB-BACK^ and KLHL9^BTB-BACK^ homology models, and these were superimposed onto KLHL3^BTB-BACK^ in the Cul3 complex structure. No insertions or deletions are present near Keap1 C151 and KLHL9 L95 relative to KLHL3, and the backbones of the three BTB-BACK domains were in excellent agreement in the areas near the mutation sites.

## Results and Discussion

### Crystal Structure of the KLHL3/Cul3 Complex

**Figure 1:**
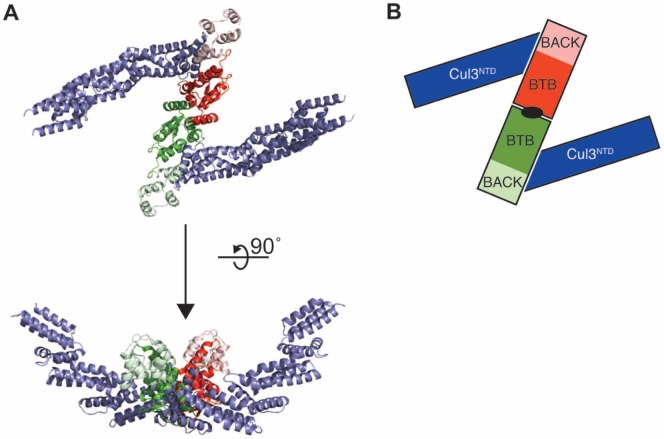
Crystal structure of the 2∶2 KLHL3^BTB-BACK^/Cul3^NTD^ complex. (**A**) The KLHL3^BTB-BACK^ homodimer (green and red) binds to two Cul3^NTD^ chains (blue). The KLHL3 BTB domains are in bold colors, and the BACK domains are in lighter colors. (**B**) Schematic of the 2∶2 complex. The view is along the BTB dimerization axis, indicated in black.

The structure of human KLHL3^BTB-BACK^/Cul3^NTD^ complex reveals a 2∶2 complex, with two chains of Cul3^NTD^ bound independently to two equivalent and non-overlapping surfaces of a KLHL3^BTB-BACK^ homodimer (Figure 1, Table 1). The Cul3^NTD^ chains are positioned to form a cup enclosing the space where the substrate-binding Kelch domains of KLHL3 are predicted to be located. These findings are consistent with suprafacial arrangements [49] observed in two other dimeric Cul3 complexes: SPOP^BTB^/Cul3^NTD^ [9] and KLHL11^BTB-BACK/^Cul3^NTD^ [34]. In solution, KLHL3^BTB-BACK^ elutes as a dimer by size exclusion chromatography, Cul3^NTD^ elutes as a monomer, and an equimolar mixture of the two proteins elutes as a single peak with the expected size for a 2∶2 complex (Figure S2).

**Table 1:** X-ray Data Collection and Refinement Statistics.

Data Collection	
Space Group	C 2 2 2_1_
Cell dimensionsa, b, c (Å)α, β, γ (°)	40.8, 228.7, 240.090, 90, 90
Wavelength (Å)	0.979
Resolution (Å)	20–3.5
Highest resolution shell (Å)	3.64–3.50
Total reflections	49265
Unique reflections	13785 (1286)
I/σ (I)	10.0 (3.1)
Rsym (%)	12.3 (53)
Completeness (%)	94.4 (89.7)
Multiplicity	3.6
**Refinement**	
Number of reflections in working set	12406
Number of reflections in test set	1379
Rwork (%)	0.24 (0.29)
Rfree_10%_ (%)	0.28 (0.31)
Average B -factors (Å^2^)	45
Number of atoms	4339
Protein residues	538
RMSD from ideal	
Bond lengths (Å)	0.004
Bond angles (°)	0.86
Ramachandran analysisPreferred/Allowed/Outlier (%)	96.0/3.8/0.2

Statistics for the highest-resolution shell are shown in parentheses.

The structure of the BTB domain of KLHL3 resembles previously solved long-form BTB domains [50], and consists of a three-stranded β-sheet flanked by seven α-helices. The first two helices form the majority of the dimerization interface [50], [51]. The strand-exchanged interchain β-sheet involving an N-terminal “β1″ strand has been observed in many long-form BTB domain dimers [50], but is not present in the KLHL3 dimer. This element is not universally present in every BTB dimer [51]. We designate the first beta strand in the KLHL3 BTB domain as β2 in order to remain consistent with the naming convention used in other BTB structures [50]–[53]. A short turn of α-helix is present in the loop between α3 and β4, and we designate this helix as α3.1, since this structural element is not observed in unliganded BTB structures (see below). The BACK domain of KLHL3 is similar to the BACK domains from KLHL11 [34] and Gigaxonin [54], with no evidence for the BACK-mediated higher order structures as seen with SPOP^BTB-BACK^ in solution [9]. The KLHL3 BACK domain spans residues 150–276 and is expected to consist four sets of helical hairpins, however only the first 5 helices (two and one half hairpins) could be reliably modeled into the electron density maps. The first BACK hairpin, residues 150–176, corresponds to the 3-box region [9], [54]. Cul3^NTD^ is made up of three, 5-helix cullin repeats similar to those previously reported in Cul1 [4], Cul4A [5], Cul4B [55], SPOP/Cul3 [9], and KLHL11/Cul3 [34].

### KLHL3/Cul3 Interaction Interface

**Figure 2:**
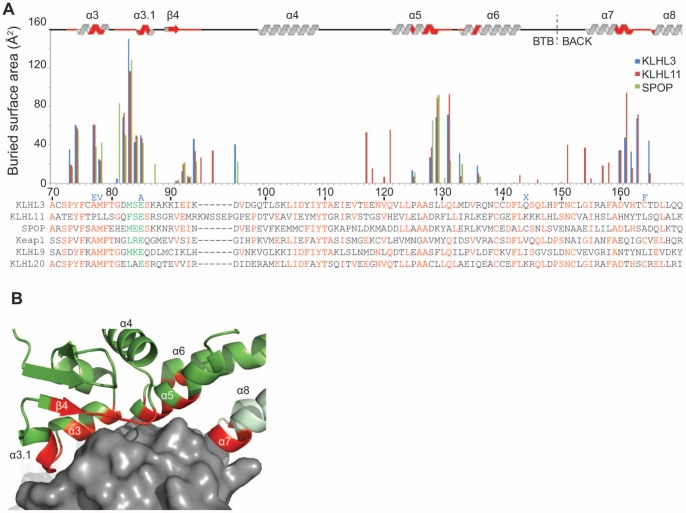
The KLHL3/Cul3 interface. (**A**) Residue-based buried surface area for KLHL3 (PDB ID 4HXI), KLHL11 (PDB ID 4AP2), and SPOP (PDB ID 4EOZ) when bound to Cul3. The multiple sequence alignment of Cul3 interacting proteins is colored by conservation, with the φ-x-E motif residues in green. Mutations identified in PHAII are indicated in blue above the KLHL3 sequence. “X” indicates a stop codon. (**B**) KLHL3 is shown in green ribbons with residue positions in contact with Cul3 highlighted in red. Cul3 is shown as a grey surface. The KLHL3 BACK domain is colored light green.

**Figure 3:**
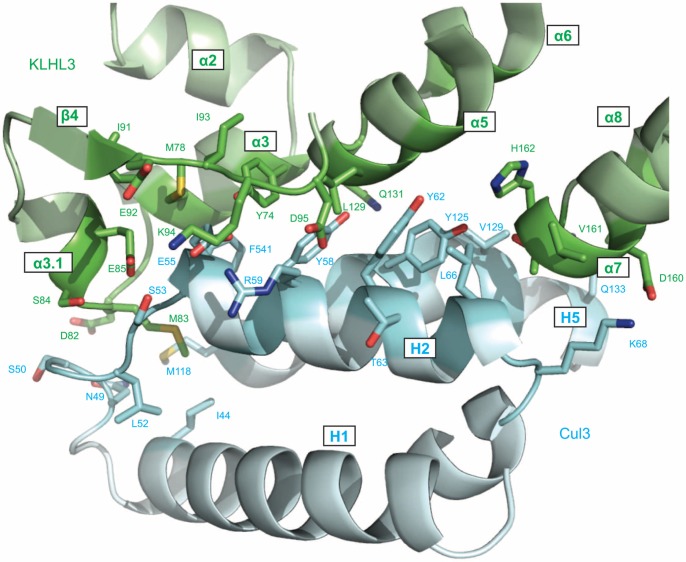
Details of the KLHL3^BTB-BACK/^Cul3^NTD^ interface. The side chains of the residues at the protein-protein interface are indicated.

The Cul3 binding interface on KLHL3 consists mostly of surfaces in the BTB domain, with important contributions from the BACK domain. Four distinct regions of KLHL3 make up the Cul3 binding region: i) helices α3 and α3.1 in the α3/β4 loop, ii) β4 and residues in the β4/α4 loop, iii) α5 and the α5/α6 loop, and iv) α7 and the α7/α8 loop (Figures 2, 3). The first three regions are in the BTB domain and the fourth is in the 3-box element of the BACK domain. With the exception of region ii, all of the KLHL3 binding elements correspond the C-terminal end of an α-helix followed by several of the following loop residues (Figure 2). Overall, the KLHL3^BTB-BACK^/Cul3^NTD^ binding interface consists of 25 residues from KLHL3 and 26 residues from Cul3, and collectively bury 1066 Å^2^ of surface area. Approximately 80% of the KLHL3 binding surface can be attributed the BTB domain region, with the remainder coming from the BACK domain. This is consistent with findings from the SPOP^BTB^/Cul3^NTD^ crystal structure, however in that case, the contributions from the 3-box/BACK region were deduced from a predicted model [9]. Solution studies showed that the SPOP BACK domain was required for full binding to Cul3: the SPOP^BTB^/Cul3^NTD^ dissociation constant was 1.0 mM, but the SPOP^BTB-BACK^/Cul3^NTD^ dissociation constant was 13 nM as measured by ITC [9].

A multiple sequence alignment of BTB-BACK-Kelch proteins reveals that the α3/α3.1 region is relatively well conserved, while other Cul3 binding regions have lower sequence similarity (Figure 2). As discussed in a later section, PHAII mutations at positions A77, M78, and E85 are found in this location [22], foreshadowing the impact of these mutations on Cul3 binding. The φ-x-E motif that was first identified in the SPOP^BTB^/Cul3^NTD^ structure [9] is preserved in the KLHL3, where the large hydrophobic residue designated by φ is M83, the charged/polar “x” residue is S84, and the conserved glutamate is E85. The φ residue buries the most surface area of any amino acid in the three known BTB/Cul3 structures (Figure 2), and is nested in a deep pocket in Cul3 that is formed between the H1–H2 loop and helix H5 (Figure 3) [9]. Residue E85 is located in the short α3.1 helix that is disordered in several uncomplexed BTB-BACK structures including KLHL11 [34] and Gigaxonin [54]. Cul3 binding induces an ordering of this region between α3 and β4, including the formation of α3.1, in all three BTB/Cul3 complexes. The added buried surface area in KLHL11 in the regions preceding α4, α5 and α7 (Figure 2) is due to contacts with additional N-terminal residues in the Cul3 construct used in reference 34, which were not present in our construct. While this region of Cul3 affects the affinity of complex in KLHL11, it does not have an effect on the overall geometry of the Cul3 assembly, as shown by the similarity of the PDB structures 4AP2 and 4APF [34].

**Figure 4:**
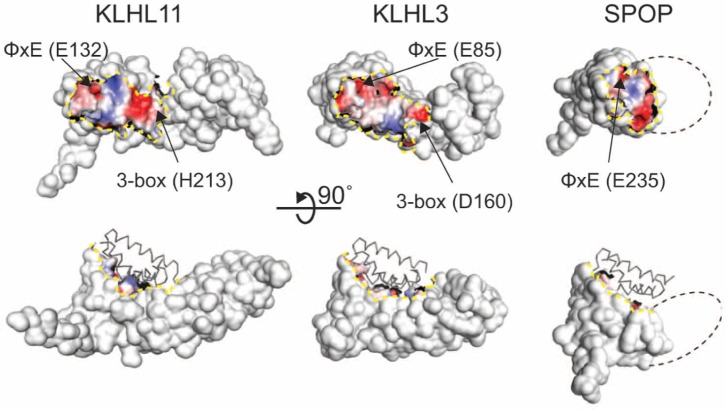
Cul3 binding surfaces of BTB-BACK proteins. The solvent-accessible surfaces of KLHL3^BTB-BACK^, KLHL11^BTB-BACK^ and SPOP^BTB^ are shown, with the Cul3-contacting region colored by the electrostatic potential, as indicated. A dashed yellow line delineates the Cul3-contacting region. The dashed black line indicates the approximate region where the BACK domain would be found in the SPOP structure. The three proteins are in similar orientations in the two views. Cul3 is shown in Cα trace in the lower set of structures.

The overall shape of the Cul3-interacting surface formed by the BTB and BACK regions is fairly consistent, however, the characteristics of these surfaces are remarkably varied (Figure 4). For example, the electrostatic potential in the Cul3 binding region of KLHL3, KLHL11 and SPOP does not reveal a consistent pattern apart from the electronegative region near the conserved φ-x-E motif, reflecting the lower sequence identity in regions ii, iii and iv. The Cul3 helix H2 makes several important contributions to the interaction, and the hydrophobic Cul3 residues F54, Y58, Y62 and L66 lie approximately along one side of recognition helix H2 and forms contacts with regions i, iii and iv from KLHL3 (Figures 3 and S1) [9].

### Overall Architecture of Dimeric CRL3 Complexes

Overall, the individual subunits in the three available BTB/Cul3 complexes (KLHL3^BTB-BACK^/Cul3^NTD^ (this work), SPOP^BTB^/Cul3^NTD^ [9] and KLHL11^BTB-BACK^/Cul3^NTD^ (PDB ID 4AP2)) are similar. The three Cul3 chains can be superposed with an average Cα RMSD of 0.6 Å, and the three BTB domains can be superposed with an average Cα RMSD of 1.2 Å The BACK domain of KLHL3 and KLHL11 are less similar, and superpose with a Cα RMSD of 2.2 Å.

**Figure 5:**
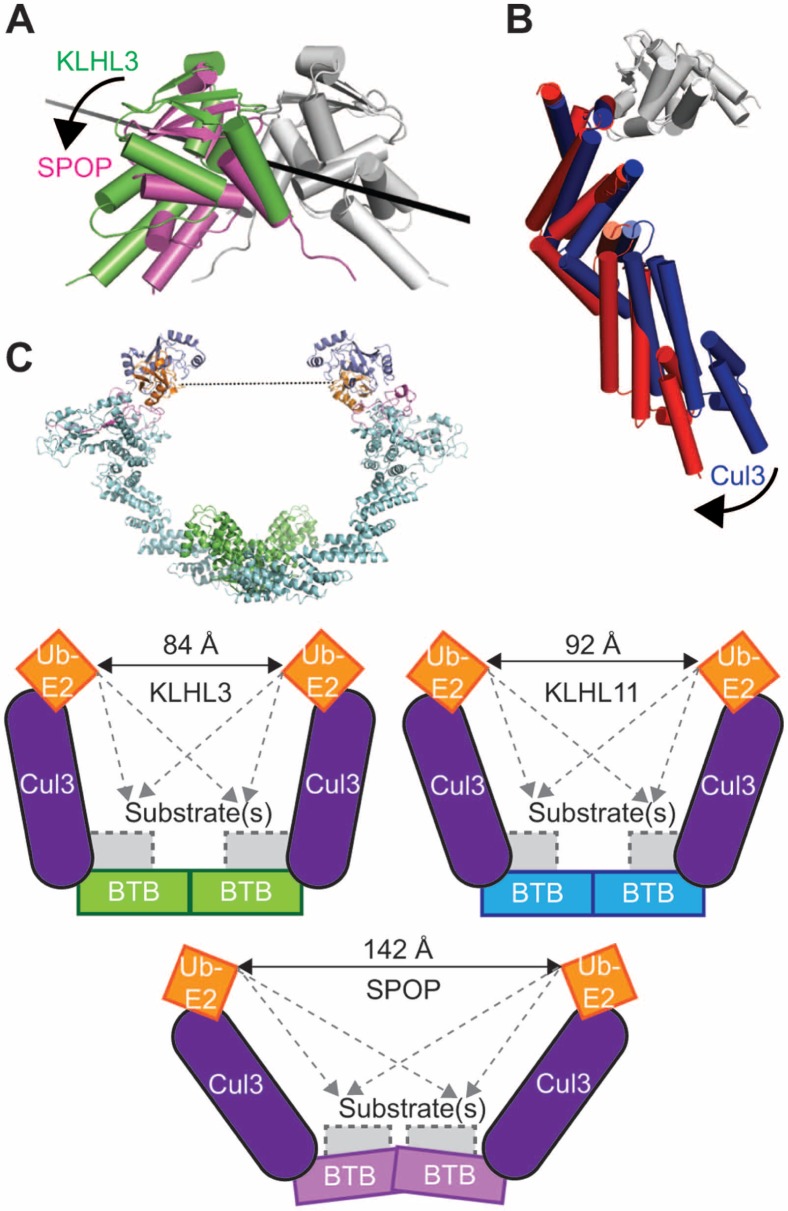
Differences in the quaternary structures of BTB/Cul3 complexes. (**A**) Comparison of the dimerization interfaces of the KLHL3 and SPOP BTB domains. A single BTB chain from KLHL3 and SPOP was superposed (shown in white and grey), resulting in a misalignment of the partner BTB chains. The second chain in the KLHL3 BTB dimer is shown in green, and the second chain of the SPOP BTB dimer is shown in magenta. The axis of rotation is shown as a black line and is approximately normal to the BTB dimerization axis. (**B**) Comparison of Cul3 chains from KLHL3 (blue) and SPOP (red) after aligning BTB domains (**C**) Model and schematics of fully assembled BTB/Cul3/E2/Ubiquitin complexes. The distances between the E2 ubiquitin conjugation sites are shown as solid arrows, and the dashed grey arrows illustrate distances to substrate binding locations. They grey regions indicate the substrate-binding Kelch domains in KLHL3 and KLHL11, and the MATH domain in SPOP.

There are larger differences at the quaternary level, and we observe small but significant differences at the interchain interfaces. Because of the similarities of the structures at the single chain level, we measured these differences as rigid body motions between the subunits. First, at the level of the BTB/BTB interfaces, there is a relative rotation of 4° between the BTB dimer interfaces of KLHL3 and KLHL11, and a much larger 14° rotation between the dimers from SPOP and KLHL3 (Figure 5A). Similar changes have been observed in the BTB interfaces of domains from BTB-Zinc finger transcription factors [51]. At the BTB/Cul3 level, a superposition of the three BTB domains reveals rotations of the Cul3 subunits by 3.1° and 11.5° in the KLHL11 and SPOP complexes, respectively, relative to the KLHL3 complex (Figure 5B).

The cumulative result of these interface differences may result in larger changes in the position of the ubiquitin-linked E2 in intact CRL3 complexes (Figure 5C). The structures of Cul1/Skp1 [4], CBL-UBCH7 [46] and UbcH5b-ubiquitin [56] were used to model the C terminus of Cul3, Skp1 and an E2/Ubiquitin complex. Assuming a rigid association from the Cul3 N-terminal region to the E2-ubiquitin region, the alterations in the BTB/BTB and BTB/Cul3 interfaces may result in a wider opening between the E2 regions in SPOP relative to KLHL3 or KLHL11. The relevance of this modeling study on the effects of substrate ubiquitination are is not clear, however, since activation of CRLs by NEDD8 results in a open, dynamic state for the complexes with conformational variability of the cullin C-terminal domain/Rbx1-tethered E2 relative to the rest of the complex [57], [58]. This allows the E2/ubiquitin moiety to sample a larger region of space, possibly erasing any differences that we deduce for the rigid complexes.

### PHAII Mutations and Cul3 Binding

**Figure 6:**
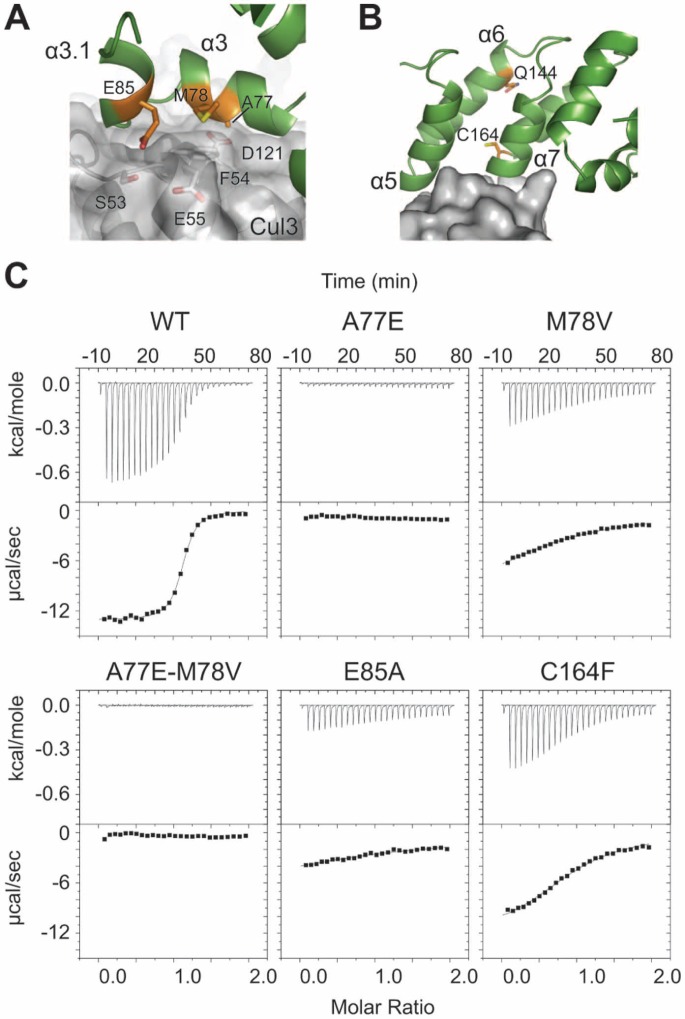
KLHL3 BTB-BACK domain mutations in PHAII. KLHL3 is shown as a green ribbon and Cul3 is shown as a transparent grey surface. Relevant PHAII mutations are colored orange. (**A**) KLHL3 residues A77, M78 and E85 are in direct contact with Cul3. (**B**) KLHL3 residue C164 is near, but not in direct contact with Cul3. Q144 indicates the location of the PHAII nonsense mutation. (**C**) ITC data and binding isotherms for the KLHL3^BTB-BACK^/Cul3^NTD^ interaction for wild-type and KLHL3 PHAII mutants.

Mutations in KLHL3 have been found in patients with PHAII [21], [22], and while a majority of these are found in the Kelch domain of the protein, mutations in the BTB and BACK domain have also been identified [22]. We used ITC to measure the affinity between KLHL3^BTB-BACK^ and Cul3^NTD^ and tested all of the identified missense mutations in the KLHL3 BTB and BACK domains from the study by Boyden *et al.* [22] (Figure 6). We measured a K_d_ of 108±8 nM and a stoichiometry of 1∶1 for the association between wild-type KLHL3^BTB-BACK^ and Cul3^NTD^. PHAII mutations A77E and M78V map to KLHL3 helix α3 and mutation E85A localizes to the φ-x-E motif in helix α3.1 (Figure 2). In the wild-type protein, all three of these positions are in direct contact with Cul3 (Figure 2 and 6A). All three of these substitutions severely disrupted Cul3 binding and K_d_ values could not be determined for these proteins (Figure 6C). The C164F mutation was the least disruptive and produced a K_d_ of 4.1±0.4 µM, a 37 fold decrease in affinity. C164 is near the interaction interface, but is not in direct contact with Cul3. This residue is located in the α7/α8 helical hairpin of the 3-box/BACK domain and is flanked by several residues that interact directly with Cul3 (Figure 2 and 6B). A mutation of this cysteine to a bulky phenylalanine may weaken the association by altering the conformation of the neighboring residues.

All of the PHAII point mutants could be produced as stable, well-folded proteins with biochemical properties that were indistinguishable from those of wild-type KLHL3^BTB-BACK^. *In vitro*, we found that the KLHL3^BTB-BACK^ point mutations were properly folded and dimeric by size exclusion chromatography, but had reduced affinity for Cul3. In homozygotes, these mutant proteins would preserve the bivalent binding of substrate through the two C-terminal Kelch motifs, but with reduced or no affinity for Cul3/Rbx1/E2∼Ub. In contrast, the expected protein produced by the Q144STOP mutation [22] would generate a truncated protein with an intact BTB domain but with no BACK or Kelch motifs (Figure 2 and 6B). This KLHL3^BTB^ chain would be competent for BTB-dimerization, but would lack the domains required for Cul3 and substrate binding. In heterozygotes, we propose that BTB-driven heterodimers could form between the missense or truncated copy of the protein and the wild-type copy of the protein.

Overall, we conclude that the KLHL3 BTB-BACK domain mutations found in PHAII patients disrupt Cul3 binding and likely reduces or abrogates the ubiquitination of substrate protein(s) bound to the Kelch domain of KLHL3. In PHAII, the relevant KLHL3 substrate appears to be the NaCl cotransporter (NCC) [21]–[23]. Failure to regulate NCC levels and subcellular localization via a functional CRL3^KLHL3^ complex may result in an overabundance and increased activity of NCC, disrupting electrolyte homeostasis and contributing to the hypertensive phenotype.

### Key residues in other BTB-BACK-Kelch Proteins

We modeled the Cul3 complex of Keap1, a redox stress sensing BTB-BACK-Kelch protein, based on our KLHL3^BTB-BACK^/Cul3^NTD^ structure. Keap1 and KLHL3 are near-neighbors in sequence space and share 33% sequence identity. This makes KLHL3 a preferred template for modeling relative to KLHL11 or Gigaxonin, which only share 19% and 21% identity to Keap1, respectively. Keap1 residue C151 (equivalent to residue N119 in KLHL3, Figure 2A) has been shown to be covalently modified by electrophiles, resulting in the disruption of Cul3 binding [11], [59]–[61]. Our model places Keap1 C151 in a loop preceding BTB helix α5, in the vicinity of Cul3, but not in direct contact (Figure S3A). By similarity to KLHL3, we predict that C151 in a well-ordered region of Keap1 and is solvent accessible. Thus, it is reasonable to assume that electrophile adduction to C151 would affect the position of nearby residues, producing a conformational change that could be transmitted to the α5 and α5/α6 loop region which is in direct contact with Cul3 (Figure S3B).

In the case of KLHL9, a L95F mutation is associated with an autosomal dominant distal myopathy [13]. This position is equivalent to KLHL3 residue I93, a residue that is partly buried in the BTB domain and also partly exposed at the Cul3 binding surface. Thus, it is thus very likely that substitutions at this position in KLHL9 would perturb the Cul3 binding interaction and affect substrate ubiquitination.

### Conclusions

The crystal structure of the BTB-BACK domains of KLHL3 in complex with an N terminal domain of Cul3 reveals the basis for the association between these two proteins. The BTB dimer generates a 2∶2 complex in which two Cul3 chains bind independently to the BTB-BACK regions of each KLHL3 subunit. Several hypertension disease mutations in KLHL3 map to the Cul3-binding region and disrupt complex formation. These results provide a molecular basis for understanding the defects in diseases involving CRL3 complexes.

## Supporting Information

Figure S1 **Electron density maps.** **(A)** As a test for the molecular replacement solution, an |Fo-Fc| map was calculated from a molecular replacement model which did not include Cul3 helix H2 and contoured at 2σ. **(B)** An |Fo-Fc| omit map was calculated based on the final refined structure, and shows good agreement between the model and the density.
(EPS)

Figure S2 **Size exclusion chromatography.** Elution profiles are shown for KLHL3^BTB-BACK^, Cul3^NTD^ and an equimolar mixture of the two proteins. Size standards are indicated. The calculated MW of a Cul3^NTD^ monomer, a KLHL3^BTB-BACK^ homodimer and a KLHL3^BTB-BACK^/Cul3^NTD^ 2∶2 complex are 42 kDa, 63 kDa and 148 kDa, respectively. The slightly larger apparent molecular weights for KLHL3^BTB-BACK^, Cul3^NTD^ and the complex are most likely due to the non-spherical shape of the proteins. V_o_ indicates the void volume of the column.
(EPS)

Figure S3 **Models of Keap1 and KLHL9.** The BTB domains are shown as green ribbons and Cul3 is shown as a grey surface. (**A**) The position of the Keap1 electrophile-sensitive residue C151 is shown in orange. (**B**) The position of KLHL9 L95 is shown in orange. See the main text for details.
(EPS)

## References

[1] Petroski MD, Deshaies RJ (2005) Function and regulation of cullin-RING ubiquitin ligases. Nat Rev Mol Cell Biol 6: 9–20. 15688063 10.1038/nrm1547

[2] Sarikas A, Hartmann T, Pan ZQ (2011) The cullin protein family. Genome Biol 12: 220. 21554755 10.1186/gb-2011-12-4-220 PMC3218854

[3] Furukawa M, He YJ, Borchers C, Xiong Y (2003) Targeting of protein ubiquitination by BTB-Cullin 3-Roc1 ubiquitin ligases. Nat Cell Biol 5: 1001–1007. 14528312 10.1038/ncb1056

[4] Zheng N, Schulman BA, Song L, Miller JJ, Jeffrey PD, et al (2002) Structure of the Cul1-Rbx1-Skp1-F boxSkp2 SCF ubiquitin ligase complex. Nature 416: 703–709. 11961546 10.1038/416703a

[5] Angers S, Li T, Yi X, MacCoss MJ, Moon RT, et al (2006) Molecular architecture and assembly of the DDB1-CUL4A ubiquitin ligase machinery. Nature 443: 590–593. 16964240 10.1038/nature05175

[6] Xu L, Wei Y, Reboul J, Vaglio P, Shin T-H, et al (2003) BTB proteins are substrate-specific adaptors in an SCF-like modular ubiquitin ligase containing CUL-3. Nature 425: 316–321. 13679922 10.1038/nature01985

[7] Geyer R, Wee S, Anderson S, Yates J, Wolf DA (2003) BTB/POZ domain proteins are putative substrate adaptors for cullin 3 ubiquitin ligases. Mol Cell 12: 783–790. 14527422 10.1016/s1097-2765(03)00341-1

[8] Pintard L, Willems A, Peter M (2004) Cullin-based ubiquitin ligases: Cul3-BTB complexes join the family. EMBO J 23: 1681–1687. 15071497 10.1038/sj.emboj.7600186 PMC394240

[9] Errington WJ, Khan MQ, Bueler SA, Rubinstein JL, Chakrabartty A, Privé GG (2012) Adaptor Protein Self-Assembly Drives the Control of a Cullin-RING Ubiquitin Ligase. Structure 20: 1141–1153. 22632832 10.1016/j.str.2012.04.009

[10] Itoh K, Wakabayashi N, Katoh Y, Ishii T, Igarashi K, et al (1999) Keap1 represses nuclear activation of antioxidant responsive elements by Nrf2 through binding to the amino-terminal Neh2 domain. Genes Dev 13: 76–86. 9887101 10.1101/gad.13.1.76 PMC316370

[11] McMahon M, Lamont DJ, Beattie KA, Hayes JD (2010) Keap1 perceives stress via three sensors for the endogenous signaling molecules nitric oxide, zinc, and alkenals. Proc Natl Acad Sci U S A 107: 18838–18843. 20956331 10.1073/pnas.1007387107 PMC2973898

[12] Taguchi K, Motohashi H, Yamamoto M (2011) Molecular mechanisms of the Keap1–Nrf2 pathway in stress response and cancer evolution. Genes Cells 16: 123–140. 21251164 10.1111/j.1365-2443.2010.01473.x

[13] Cirak S, von Deimling F, Sachdev S, Errington WJ, Herrmann R, et al (2010) Kelch-like homologue 9 mutation is associated with an early onset autosomal dominant distal myopathy. Brain 133: 2123–2135. 20554658 10.1093/brain/awq108 PMC2892937

[14] Rondou P, Skieterska K, Packeu A, Lintermans B, Vanhoenacker P, et al (2010) KLHL12-mediated ubiquitination of the dopamine D4 receptor does not target the receptor for degradation. Cell Signal 22: 900–913. 20100572 10.1016/j.cellsig.2010.01.014

[15] Funato Y, Terabayashi T, Sakamoto R, Okuzaki D, Ichise H, et al (2010) Nucleoredoxin sustains Wnt/β-catenin signaling by retaining a pool of inactive dishevelled protein. Curr Biol 20: 1945–1952. 20970343 10.1016/j.cub.2010.09.065

[16] Angers S, Thorpe CJ, Biechele TL, Goldenberg SJ, Zheng N, et al (2006) The KLHL12-Cullin-3 ubiquitin ligase negatively regulates the Wnt-beta-catenin pathway by targeting Dishevelled for degradation. Nat Cell Biol 8: 348–357. 16547521 10.1038/ncb1381

[17] Jin L, Pahuja KB, Wickliffe KE, Gorur A, Baumgärtel C, et al (2012) Ubiquitin-dependent regulation of COPII coat size and function. Nature 482: 495–500. 22358839 10.1038/nature10822 PMC3292188

[18] Yuan WC, Lee YR, Huang SF, Lin YM, Chen TY, et al (2011) A Cullin3-KLHL20 Ubiquitin ligase-dependent pathway targets PML to potentiate HIF-1 signaling and prostate cancer progression. Cancer Cell 20: 214–228. 21840486 10.1016/j.ccr.2011.07.008

[19] Higashimura Y, Terai T, Yamaji R, Mitani T, Ogawa M, et al (2011) Kelch-like 20 up-regulates the expression of hypoxia-inducible factor-2α through hypoxia- and von Hippel-Lindau tumor suppressor protein-independent regulatory mechanisms. Biochem Biophys Res Commun 413: 201–205. 21888897 10.1016/j.bbrc.2011.08.058

[20] Lai F, Orelli BJ, Till BG, Godley LA, Fernald AA, et al (2000) Molecular characterization of KLHL3, a human homologue of the Drosophila kelch gene. Genomics 66: 65–75. 10843806 10.1006/geno.2000.6181

[21] Louis-Dit-Picard H, Barc J, Trujillano D, Miserey-Lenkei S, Bouatia-Naji N, et al (2012) KLHL3 mutations cause familial hyperkalemic hypertension by impairing ion transport in the distal nephron. Nat Genet 44: 456–460. 22406640 10.1038/ng.2218

[22] Boyden LM, Choi M, Choate KA, Nelson-Williams CJ, Farhi A, et al (2012) Mutations in kelch-like 3 and cullin 3 cause hypertension and electrolyte abnormalities. Nature 482: 98–102. 22266938 10.1038/nature10814 PMC3278668

[23] Wilson FH, Kahle KT, Sabath E, Lalioti MD, Rapson AK, et al (2003) Molecular pathogenesis of inherited hypertension with hyperkalemia: the Na-Cl cotransporter is inhibited by wild-type but not mutant WNK4. Proc Natl Acad Sci U S A 100: 680–684. 12515852 10.1073/pnas.242735399 PMC141056

[24] Wilson FH, Disse-Nicodème S, Choate KA, Ishikawa K, Nelson-Williams C, et al (2001) Human hypertension caused by mutations in WNK kinases. Science 293: 1107–1112. 11498583 10.1126/science.1062844

[25] Choi M, Scholl UI, Yue P, Björklund P, Zhao B, et al (2011) K+ channel mutations in adrenal aldosterone-producing adenomas and hereditary hypertension. Science 331: 768–772. 21311022 10.1126/science.1198785 PMC3371087

[26] Lifton RP, Gharavi AG, Geller DS (2001) Molecular Mechanisms of Human Hypertension. Cell 104: 545–556. 11239411 10.1016/s0092-8674(01)00241-0

[27] Meneton P, Jeunemaitre X, de Wardener HE, MacGregor GA (2005) Links between dietary salt intake, renal salt handling, blood pressure, and cardiovascular diseases. Physiol Rev 85: 679–715. 15788708 10.1152/physrev.00056.2003

[28] Piper RC, Luzio JP (2007) Ubiquitin-dependent sorting of integral membrane proteins for degradation in lysosomes. Curr Opin Cell Biol 19: 459–465. 17689064 10.1016/j.ceb.2007.07.002 PMC2046217

[29] Schwarz LA, Hall BJ, Patrick GN (2010) Activity-dependent ubiquitination of GluA1 mediates a distinct AMPA receptor endocytosis and sorting pathway. J Neurosci 30: 16718–16729. 21148011 10.1523/JNEUROSCI.3686-10.2010 PMC3079366

[30] Fykerud TA, Kjenseth A, Schink KO, Sirnes S, Bruun J, et al (2012) Smad ubiquitination regulatory factor-2 controls gap junction intercellular communication by modulating endocytosis and degradation of connexin43. J Cell Sci 125: 3966–3976. 22623726 10.1242/jcs.093500

[31] Guo J, Wang T, Li X, Shallow H, Yang T, et al (2012) Cell surface expression of human ether-a-go-go-related gene (hERG) channels is regulated by caveolin-3 protein via the ubiquitin ligase Nedd4–2. J Biol Chem 287: 33132–33141. 22879586 10.1074/jbc.M112.389643 PMC3460420

[32] Aikawa Y (2012) Rabex-5 protein regulates the endocytic trafficking pathway of ubiquitinated neural cell adhesion molecule L1. J Biol Chem 287: 32312–32323. 22846990 10.1074/jbc.M112.374322 PMC3463310

[33] Ko B, Kamsteeg EJ, Cooke LL, Moddes LN, Deen PM, Hoover RS (2010) RasGRP1 stimulation enhances ubiquitination and endocytosis of the sodium-chloride cotransporter. Am J Physiol Renal Physiol 299: F300–F309. 20392800 10.1152/ajprenal.00441.2009 PMC2928521

[34] Canning P, Cooper CD, Krojer T, Murray JW, Pike AC, et al (2013) Structural basis for Cul3 assembly with the BTB-Kelch family of E3 ubiquitin ligases. J Biol Chem. Available: http://www.jbc.org/cgi/doi/10.1074/jbc.M112.437996. Accessed 01 March 2013. 10.1074/jbc.M112.437996 PMC3597819 23349464

[35] Mooij WT, Mitsiki E, Perrakis A (2009) ProteinCCD: enabling the design of protein truncation constructs for expression and crystallization experiments. Nucleic Acids Res 37: W402–405. 19395596 10.1093/nar/gkp256 PMC2703965

[36] Eschenfeldt WH, Stols L, Millard CS, Joachimiak A, Donnelly MI (2009) A family of LIC vectors for high-throughput cloning and purification of proteins. Methods Mol Biol 498: 105–115. 18988021 10.1007/978-1-59745-196-3_7 PMC2771622

[37] Goldschmidt L, Cooper DR, Derewenda ZS, Eisenberg D (2007) Toward rational protein crystallization: A Web server for the design of crystallizable protein variants. Protein Sci 16: 1569–1576. 17656576 10.1110/ps.072914007 PMC2203352

[38] Otwinowski Z, Minor W (1997) Processing of X-ray Diffraction Data Collected in Oscillation Mode. Method Enzymol 276: 307–326. 10.1016/S0076-6879(97)76066-X 27754618

[39] Minor W, Cymborowski M, Otwinowski Z, Chruszcz M (2006) HKL-3000: the integration of data reduction and structure solution–from diffraction images to an initial model in minutes. Acta Crystallogr D Biol Crystallogr 62: 859–866. 16855301 10.1107/S0907444906019949

[40] Adams PD, Afonine PV, Bunkóczi G, Chen VB, Davis IW, et al (2010) PHENIX: a comprehensive Python-based system for macromolecular structure solution. Acta Crystallogr D Biol Crystallogr 66: 213–221. 20124702 10.1107/S0907444909052925 PMC2815670

[41] Emsley P, Lohkamp B, Scott WG, Cowtan K (2010) Features and development of Coot. Acta Crystallogr D Biol Crystallogr 66: 486–501. 20383002 10.1107/S0907444910007493 PMC2852313

[42] Schrödinger LLC. The PyMOL Molecular Graphics System.

[43] Krissinel E, Henrick K (2007) Inference of macromolecular assemblies from crystalline state. J Mol Biol 372: 774–797. 17681537 10.1016/j.jmb.2007.05.022

[44] Dietmann S, Park J, Notredame C, Heger A, Lappe M, et al (2001) A fully automatic evolutionary classification of protein folds: Dali Domain Dictionary version 3. Nucleic Acids Res 29: 55–57. 11125048 10.1093/nar/29.1.55 PMC29815

[45] Holm L, Sander C (1993) Protein structure comparison by alignment of distance matrices. J Mol Biol 233: 123–138. 8377180 10.1006/jmbi.1993.1489

[46] Zheng N, Wang P, Jeffrey PD, Pavletich NP (2000) Structure of a c-Cbl-UbcH7 complex: RING domain function in ubiquitin-protein ligases. Cell 102: 533–539. 10966114 10.1016/s0092-8674(00)00057-x

[47] Hamilton KS, Ellison MJ, Barber KR, Williams RS, Huzil JT, et al (2001) Structure of a conjugating enzyme-ubiquitin thiolester intermediate reveals a novel role for the ubiquitin tail. Structure 10: 897–904. 10.1016/s0969-2126(01)00657-8 11591345

[48] Roy A, Kucukural A, Zhang Y (2010) I-TASSER: a unified platform for automated protein structure and function prediction. Nat Protoc 5: 725–738. 20360767 10.1038/nprot.2010.5 PMC2849174

[49] Tang X, Orlicky S, Lin Z, Willems A, Neculai D, et al (2007) Suprafacial orientation of the SCFCdc4 dimer accommodates multiple geometries for substrate ubiquitination. Cell 129: 1165–1176. 17574027 10.1016/j.cell.2007.04.042

[50] Stogios PJ, Downs GS, Jauhal JJS, Nandra SK, Privé GG (2005) Sequence and structural analysis of BTB domain proteins. Genome Biol 6: R82. 16207353 10.1186/gb-2005-6-10-r82 PMC1257465

[51] Stogios PJ, Cuesta-Seijo JA, Chen L, Pomroy NC, Privé GG (2010) Insights into strand exchange in BTB domain dimers from the crystal structures of FAZF and Miz1. J Mol Biol 400: 983–997. 20493880 10.1016/j.jmb.2010.05.028

[52] Ahmad KF, Engel CK, Privé GG (1998) Crystal structure of the BTB domain from PLZF. Proc Natl Acad Sci U S A 95: 12123–12128. 9770450 10.1073/pnas.95.21.12123 PMC22795

[53] Stogios PJ, Chen L, Privé GG (2007) Crystal structure of the BTB domain from the LRF/ZBTB7 transcriptional regulator. Protein Sci 16: 336–342. 17189472 10.1110/ps.062660907 PMC2203294

[54] Zhuang M, Calabrese MF, Liu J, Waddell MB, Nourse A, et al (2009) Structures of SPOP-substrate complexes: insights into molecular architectures of BTB-Cul3 ubiquitin ligases. Mol Cell 36: 39–50. 19818708 10.1016/j.molcel.2009.09.022 PMC2847577

[55] Fischer ES, Scrima A, Böhm K, Matsumoto S, Lingaraju GM, et al (2011) The molecular basis of CRL4DDB2/CSA ubiquitin ligase architecture, targeting, and activation. Cell 147: 1024–1039. 22118460 10.1016/j.cell.2011.10.035

[56] Sakata E, Satoh T, Yamamoto S, Yamaguchi Y, Yagi-Utsumi M, et al (2010) Crystal structure of UbcH5b∼ubiquitin intermediate: insight into the formation of the self-assembled E2∼Ub conjugates. Structure 18: 138–147. 20152160 10.1016/j.str.2009.11.007

[57] Duda DM, Scott DC, Calabrese MF, Zimmerman ES, Zheng N, et al (2011) Structural regulation of cullin-RING ubiquitin ligase complexes. Curr Opin Struct Biol 21: 257–264. 21288713 10.1016/j.sbi.2011.01.003 PMC3151539

[58] Duda DM, Borg L, Scott DC, Hunt HW, Hammel M, et al (2008) Structural insights into NEDD8 activation of cullin-RING ligases: conformational control of conjugation. Cell 134: 995–1006. 18805092 10.1016/j.cell.2008.07.022 PMC2628631

[59] Eggler AL, Small E, Hannink M, Mesecar AD (2009) Cul3-mediated Nrf2 ubiquitination and antioxidant response element (ARE) activation are dependent on the partial molar volume at position 151 of Keap1. Biochem J 422: 171–180. 19489739 10.1042/BJ20090471 PMC3865926

[60] Hu C, Eggler AL, Mesecar AD, van Breemen RB (2011) Modification of keap1 cysteine residues by sulforaphane. Chem Res Toxicol 24: 515–521. 21391649 10.1021/tx100389r PMC3086360

[61] Hong F, Freeman ML, Liebler DC (2005) Identification of sensor cysteines in human Keap1 modified by the cancer chemopreventive agent sulforaphane. Chem Res Toxicol 18: 1917–1926. 16359182 10.1021/tx0502138

